# Genotypic and phenotypic characterization of *Escherichia coli* isolated from indigenous individuals in Malaysia

**DOI:** 10.22038/IJBMS.2022.61612.13637

**Published:** 2022-04

**Authors:** Vanitha Mariappan, Soo Tein Ngoi, Yvonne Ai Lian Lim, Romano Ngui, Kek Heng Chua, Cindy Shuan Ju Teh

**Affiliations:** 1 Centre of Toxicology and Health Risk Studies (CORE), Faculty of Health Sciences, Universiti Kebangsaan Malaysia, 50300 Kuala Lumpur, Malaysia; 2 Department of Medical Microbiology, Faculty of Medicine, Universiti Malaya, 50603 Kuala Lumpur, Malaysia; 3 Department of Parasitology, Faculty of Medicine, Universiti Malaya, 50603 Kuala Lumpur, Malaysia; 4 Centre for Malaysian Indigenous Studies (CMIS), Universiti Malaya, Kuala Lumpur, Malaysia; 5 Department of Biomedical Science, Faculty of Medicine, Universiti Malaya, 50603 Kuala Lumpur, Malaysia

**Keywords:** Genotypic, Indigenous, Malaysia, Phenotypic, Verocytotoxin (VT)-producing Escherichia coli, (VTEC)

## Abstract

**Objective(s):**

The occurrence of asymptomatic verocytotoxin (VT)-producing *Escherichia coli *(VTEC) infections among humans in recent years is posing a high risk to public health. Thus, the role of asymptomatic human carriers as a source of dissemination should not be underestimated. This study aimed to elucidate the phenotypic and genotypic characteristics of *E. coli* in the stool samples collected from indigenous individuals in Malaysia.

**Materials and Methods:**

*E. coli *strains (n=108) were isolated from stool samples obtained from 41 indigenous individuals. All strains were subjected to Repetitive Extragenic Palindromic-Polymerase Chain Reaction (REP-PCR) typing and confirmation of VTEC variants. Non-duplicate strains were selected based on REP-PCR profiles and further subjected to antimicrobial susceptibility test (AST). The genotypic and phenotypic characteristics of the strains were then correlated with the demographic data of the subjects.

**Results:**

A total of 66 REP-PCR profiles grouped in 53 clusters (F=85%) were obtained. Four genetically distinct strains were confirmed as VTEC (*eaeA*-positive). The predominant resistance was against ampicillin (34.2%), followed by trimethoprim-sulfamethoxazole (32.9%), ampicillin-sulbactam (5.5%), and ciprofloxacin (1.4%). All isolates were sensitive to amoxicillin-clavulanate, cefuroxime, ceftriaxone, imipenem, and meropenem.

**Conclusion:**

Genetically diverse *E. coli* and VTEC strains were found to colonize the intestines of the indigenous populations. This study is important for the prospective surveillance of *E. coli *among the indigenous individuals in Malaysia, especially in asymptomatic VTEC infection and antimicrobial resistance phenomenon.

## Introduction

*Escherichia coli* (*E. coli*) is one of the commensal bacterial species that are found in human and animal intestines and feces. These commensal *E. coli *strains rarely cause disease, except in immunocompromised hosts, or when gastrointestinal barriers are violated. However, only certain serotypes of *E. coli* may cause disease in both humans and animals (1).

Enterohaemorrhagic *E. coli* (EHEC), also known as Shiga-toxin producing *E. coli* (STEC), was first described as associated with pathogenicity in 1982 (1). It is an important foodborne pathogen that causes serious public health concerns as it is highly associated with severe gastrointestinal disease (1). Characteristics of EHEC infection include abdominal cramps, bloody diarrhea, as well as hemorrhagic colitis and hemolytic uremic syndrome (2). The major virulence factor of EHEC is Shiga toxins (Stx) (1, 2). The Stx family is composed of 2 major subgroups, namely Stx1 and Stx2. The organisms may express at least one subgroup of Stx. Shiga toxin was reported to be the same as verocytotoxin (VT) (3). Thus, the term can be fully interchangeable with VT nomenclatures (*i.e., *Stx1 = VT1, Stx2 = VT2). 

EHEC can also be defined by the presence of intimin, encoded by the* eae*A gene (2, 4). This protein allows EHEC to intimately attach to epithelial cells and efface microvilli in the large intestine. Strains that specifically encode the *eaeA* gene appear to colonize any intestinal site, followed by the presence of ‘attaching and effacing’ (A/E) lesions (2). The lesion in EHEC infection is characterized by destruction of microvilli, intimate attachment of the organism to the cell, and accumulation of polymerized actin beneath the site of bacterial attachment. Previous studies have shown that even if that strain of *E. coli* may produce Stx, it can be *eaeA*-negative and not associated with any disease (5-7). This has led to the use of VT-producing *E. coli* (VTEC) or STEC as a general term to name those strains that produce Stx, and the term EHEC is used only for the one that produces Stx together with the *eaeA*-positive genotype. 

In Malaysia, the native indigenous populations generally live in poor conditions, low hygiene practices, and lack of functioning toilet facilities in their houses. These factors were found to be significantly related to microbiota diversity among them compared with the major ethnic groups (Malay and Chinese) (8). Hence, many studies associated with sanitation-related diseases have been carried out over the years (9, 10). Based on the findings by Teh* et al.* (2014) asymptomatic VTEC infection has been observed among the indigenous community, and most of the asymptomatic infections occurred in those aged below 15 years (11). Thus, the role of asymptomatic human carriers as a source of dissemination should not be underestimated.  

National surveillance for antimicrobial resistance (AMR) among *E. coli* in Malaysia has observed relatively high rates of resistance to commonly prescribed antibiotics such as ampicillin (AMP) (66.2–67.0%), ampicillin-sulbactam (SAM) (22.9–26.6%), cefuroxime (CXM) (22.6–25.9%), ciprofloxacin (CIP) (23.2–24.9%), and trimethoprim-sulfamethoxazole (SXT) (38.9–41.5%) (12). Furthermore, multiple-drug resistant (MDR) *E. coli *has also been increasingly reported in this region (13). Additionally, the findings of Liu* et al.* (2016) regarding the emergence of a new plasmid-mediated colistin resistance gene *mcr-1 *raised great concern among researchers that the world is on the cusp of a post-antibiotic era (14). Polymyxins (colistin and polymyxin B) are known as the last line of defense against many Gram-negative bacilli. The treatment for serious infections due to highly resistant bacteria such as extended-spectrum beta-lactamase (ESBL) and carbapenemase-producing *E. coli* are usually restricted to tigecycline and colistin (15). Thus, emergence of colistin resistance is inevitable. There is also evidence that it has spread to Malaysia (16-18).

Hence, we aim to study the phenotypic and genotypic characteristics of *E. coli* in the stool samples collected from indigenous individuals in Malaysia using Repetitive Extragenic Palindromic Polymerase Chain Reaction (REP-PCR) and antimicrobial susceptibility test (AST). The strains were further characterized based on genes associated with VT production (*vtx1*, *vtx2*, *vtx2f, *and *eaeA* genes) and polymyxin resistance (*mcr-1* gene). 

## Materials and Methods

***Sample population, study sites, and ethical approval***

This study was approved by the Medical Ethics Committee of the University of Malaya Medical Centre (UMMC) (Ethics committee/IRB reference number: UMMREC 920.83). Indigenous individuals with known parasitic infections were recruited in this study. Subject recruitments were conducted at three indigenous villages, namely Kampung Gurney at Hulu Yam, Kampung Sungai Judah, and Kampung Kepau Laut at Carey Island. All three indigenous villages are located in Selangor, Malaysia. The stool sample collection procedure has also been approved by the Department of Orang Asli Development (JAKOA) with strict adherence to the Helsinki Declaration of 1975, as revised in 2000. Consent was given by the participating indigenous individuals either via signature or thumbprint before sample collection (parent’s consent was obtained for kids below 18 years of age). 

***Bacterial strains***

*E. coli* strains were isolated from the stool samples collected from the subjects, using a previously described protocol (19). In brief, 0.1 g of the stool sample was diluted (1:10) in phosphate-buffered saline and inoculated onto Oxoid^TM^ MacConkey agar plates (Fisher Scientific, Malaysia). The inoculated agar plates were then incubated aerobically at 37 °C. Following 20 hr of incubation, the plates were examined for growth and colony characteristics, where *E. coli* presented as pink colonies. The *E. coli *strains were further confirmed via polymerase chain reaction (PCR) to detect the *pho*A gene (20). The strains were stored in 25% glycerol stock cultures at -80 °C until further use. 

***Preparation of DNA template***

DNA of the *E. coli* strains was obtained through the direct boiling method as previously described by Dashti* et al. *(2009) (21). Briefly, a loopful of bacterial culture was mixed with 100 µl of sterile deionized water and the suspension was boiled at 100 °C for 5 min. Following that, the cell lysate was snapped cool for 5 min on ice and centrifuged at 10,000 g for 2 min. The supernatant was used as the DNA template for PCR analyses.

***Repetitive extragenic palindromic (REP) – PCR***

REP-PCR was performed using REP primer as previously described by Navia* et al. *(1999) (22). PCR was carried out in a total volume of 25 µl containing 2.5 mM MgCl_2_, 50 µM of each dNTP, 0.6 µM of primer, and 1.0 U *Taq* DNA polymerase (Promega, USA). The PCR cycling conditions consisted of 94 °C for 4 min (initial denaturation) followed by 35 cycles of 94 °C for 1 min, 42 °C for 1 min, 68 °C for 8 min, and a final extension of 8 min at 72 °C. The amplicons were electrophoresed on a 2% (w/v) agarose gel at 80 V for 7 hr. A 1 kb DNA marker (Thermo Scientific, USA) was used as the molecular size standard. The gels were stained with SYBR Green dye (Thermo Scientific, USA) and then visualized. 

***Analysis of genomic fingerprint patterns***

Digital images of the gels were uploaded into the genomic fingerprint analysis program BioNumerics version 7.1 (Applied Maths, Belgium) and scored for banding patterns using densitometric curve-based characterization. Band positions were normalized by using a 1 kb molecular size marker (Thermo Scientific, USA) to correct for gel irregularities from electrophoresis and allow the comparison of multiple gels. A 1% tolerance level for band matching was allowed. The similarity between REP-PCR profiles was determined using the Dice coefficient (F) and a dendrogram was generated by using the unweighted pair group method with arithmetic mean (UPGMA) algorithm.

***PCR detection of verocytotoxin-producing E. coli (VTEC) isolates and colistin-resistant genes***

PCR primer pairs targeting *vtx1 and* *vtx2 *variants*, and eaeA *and *mcr-1* genes were used in this study (4, 14, 23). For VTEC gene detection, briefly, a 50 µl reaction mixture containing 1× reaction buffer, 1.2 mM MgCl_2_, 0.2 mM each dNTPs, 50 *p*moles of each primer, 2 U of *Taq* polymerase, and ~ 50 ng of DNA template was set up for each sample. The thermal cycler was programmed as described by Paton and Paton (1998) (4). Samples were subjected to 35 PCR cycles, each consisting of 1 min of denaturation at 95 °C; 2 min of annealing at 65 °C for the first 10 cycles, decrementing to 60 °C by cycle 15; and 1.5 min of elongation at 72 °C, incrementing to 2.5 min from cycles 25 to 35. The colistin-resistant gene (*mcr-1*) detection was electrophoresed according to Liu* et al. *(2016) (14). In each PCR assay, a positive (*E. coli* strains possessing the virulence genes tested) and two negative controls (non-pathogenic *E. coli* strain ATCC25922 and no-template control) were included. The amplicons were electrophoresed on a 1% agarose gel at 110 V for an hour. A 100 bp DNA marker (Thermo Scientific, USA) was used as the molecular size standard. The gels were stained with SYBR Green dye (Thermo Scientific, USA) and then visualized.

***Antimicrobial susceptibility testing***

Seventy-three non-duplicated strains identified based on REP-PCR profile analysis were tested by disk diffusion method against nine different antimicrobial agents, namely AMP (10 µg), amoxicillin-clavulanic acid (AMC) (20 µg/ 10 µg), ceftriaxone (CRO) (30 µg), CXM (30 µg), CIP (5 µg), SXT (1.25 µg/ 23.75 µg), imipenem (IPM) (10 µg), meropenem (MEM) (10 µg), and SAM (10 µg/ 10 µg). The zone of inhibition was measured and interpreted according to 2015 Clinical and Laboratory Standards Institution guidelines (24). Each *E. coli *strain was streaked onto LB agar and incubated at 37 °C. Following overnight incubation, four or five well-isolated colonies were mixed with sterile saline in a plastic tube and 0.5 McFarland standard was obtained by checking the density of bacterial suspension using the DensiCHEK Plus instrument (bioMérieux, France). Within 15 min after adjusting the turbidity of the inoculum suspension, a sterile cotton swab was dipped into the suspension and streaked over the entire surface of Mueller-Hinton agar three times by rotating the plate approximately 60 degrees after each application to ensure an even distribution of inoculum. The disks were placed on the Mueller-Hinton agar and incubated at 35 °C for 18 to 24 hr. Following incubation, the diameters of the zones of complete inhibition were recorded. The sizes of zones of inhibition were compared with the zone-size interpretative table stated in CLSI guidelines and recorded as susceptible, intermediate, or resistant to each antibiotic tested.

***Statistical analysis***

Statistical analysis was performed to determine the relationship between variables. The correlation of each antibiotic resistance event to gender and location was calculated using the Pearson’s Chi-square test and Fisher’s exact test for smaller data sets. The odds ratio was calculated when the probability of the two variables was dependent. 

## Results

A total of 41 indigenous individuals (female, n=22; male, n=19) between the ages of 5 and 68 were recruited in this study (Supplementary Table 1). The majority of the subjects were from Kampung Gurney (n=33), followed by Kampung Sungai Judah (n=4) and Kampung Kepau Laut (n=4). Most subjects (n=39; 95%) had known parasitic infections (amoebiasis, n=3; ascariasis, n=8; giardiasis, n=2; hookworm, n=15; trichuriasis, n=35). 

**Figure 1:**
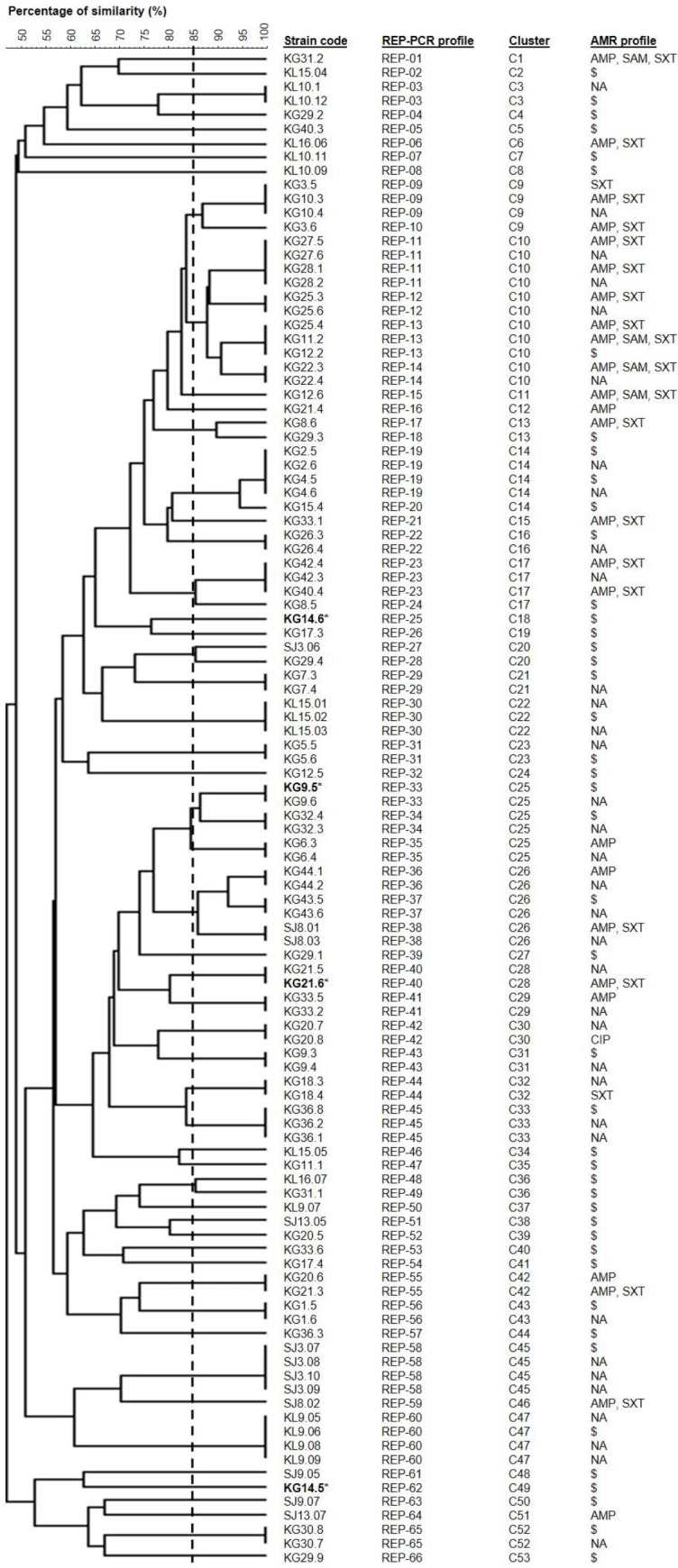
Dendrogram showing cluster analysis of Escherichia coli strains (n=108) based on REP-PCR profiles. Dice coefficient (F) and UPGMA clustering parameters at 1% position tolerance were used to construct the dendrogram. Subject populations are indicated in the first two letters of the strain codes (KG: Kampung Gurney; KL: Kampung Kepau Laut; SJ: Kampung Sungai Judah). VTEC strains are marked with an asterisk (*). The abbreviations are defined as AMR: antimicrobial resistance; AMP: ampicillin; NA: not available; SAM: ampicillin-sulbactam; SXT: trimethoprim-sulfamethoxazole. The dotted vertical line indicates similarity cut-off at F=0.85

A total of 108 *E. coli* strains were isolated from the stool samples (n=41) of the indigenous individuals and were analyzed using REP-PCR (Figure 1). The REP-PCR generated multiple DNA fragments ranging in size from 150 bp to 10,000 bp and produced genomic profiles consisting of 5 to 16 bands (Supplementary Figure 1). The genetic relationship among the strains was deduced from the dendrogram (Figure 1). For fingerprint pattern comparisons, a 1.5% optimization setting was found to give the highest similarity recognition among the strains. Cluster analysis of the genomic fingerprints of the 108 *E. coli* strains obtained through REP-PCR yielded 66 different profiles (arbitrarily designated as REP-01 to REP-66) grouped into 53 clusters (C1-C53) at a similarity coefficient of 85% (closely related) (Figure 1). 

Seventy-three different *E. coli *strains were selected for further characterization based on different REP-PCR profiles and specimen sources (i.e., isolated from different subjects). The *eaeA *gene was detected in only four strains [KG14.6, KG9.5, KG21.6, and KG 14.5 corresponding to REP-PCR profile (cluster) REP-25 (C18), REP-33 (C25), REP-40 (C28), and REP-62 (C49), respectively] (Figure 1). Among the four VTEC strains, two of them were isolated from indigenous individuals aged below 15 years of age (males) and the remaining two were from a female individual aged 29 (Supplementary Table 2). On the other hand, all strains were negative for *vtx1* and *vtx2* genes. A representative *E. coli *strain that harbored the *eaeA* gene was sent for DNA sequencing for confirmation. Consensus sequences were created from forward and reverse sequences and compared with VTEC sequences in the National Center for Biotechnology Information (NCBI) GenBank database using the Basic Local Alignment Search Tool (BLAST) server (https://blast.ncbi.nlm.nih.gov/Blast.cgi). The *E. coli *strain was confirmed to be *eaeA*-positive and BLAST analysis suggested that the strain may be in serogroup O157:H7. Similar to *vtx1* and *vtx2* genes, all 73 strains tested negative for the *mcr-1* gene (Supplementary Table 2).

The antibiotics chosen in this study were commonly prescribed for *E. coli* infections. The AMR profiles of selected *E. coli *strains (n=73) are shown in Figure 1. In summary, the AMR rates for the *E. coli* strains were; SXT 32.9%, AMP 34.2%, SAM 5.5%, and CIP 1.4%. All strains were sensitive to CXM, CRO, IPM, and MEM. However, only 1.4% of the strains showed intermediate susceptibility to AMC (Supplementary Table 2). 

The correlation between antibiotic resistance with gender and location was calculated using Pearson Chi-Square and Fisher’s exact test. There was no correlation found between the antibiotics and locations (*P*-value≥0.05). In addition, no correlation was observed between antibiotics and gender, except for SXT (*P*-value≤0.05). The odds ratio was calculated to further determine the relationship between variables. From the calculation, *E. coli *strains from male subjects showed significantly higher resistance to SXT compared with females.

## Discussion

Generally, the main goal of a strain typing study is to determine epidemiologically and genetically related strains. This information is therefore important to understand and control the spread of infectious diseases. Based on the REP-PCR analysis, complex fingerprint patterns were obtained for *E. coli* strains in this study. The dendrogram indicated the possible occurrence of bacterial transmission by sharing genetically similar (F ≥ 85%) *E. coli *strains between samples that were collected within the same area. In general, the REP-PCR patterns of *E. coli *strains from the same person were similar, thus indicating that the strains were genetically indistinguishable. Bacterial strains are known as genetically indistinguishable if their genetic fingerprinting patterns have the same number of bands and the corresponding bands are of the same size. 

*E. coli* sharing identical genotypes can be considered to represent the clonality of the strains (24). While the fingerprinting patterns for *E. coli *strain obtained from the same person were largely similar, they were not always identical. Most of the strains derived from the same area shared 85% similarity (i.e., closely related strains), while a few of them showed less than 85% genetic similarity. Bacterial strains are considered to be closely related when the genetic fingerprinting patterns differ with a single genetic event (i.e., a point mutation or an insertion or deletion of DNA). Typically, such changes may result in two to three band differences. The previous study identified that strains of some bacterial species showed two to three bands variation when they were cultured repeatedly over time or being isolated multiple times from the same patient (25). Bacterial strains that were less than 85% similar were not genetically related and could be due to at least two independent genetic events that resulted in four to six band differences. 

PCR technique is considered to be the most sensitive method to detect VTEC in fecal specimens (4). The VTEC strains identified in this study were found colonizing both adolescents and adults, contrary to the previous study done by Teh* et al.* (2014), where most of the VTEC infections were found among those aged below 15 years (11). Besides age, other factors that may affect shedding patterns of VTEC are diet, stress, and seasonal variation (6). 

Our findings showed that the *E. coli *strains found colonizing the intestines of local indigenous communities were genetically diverse and heterogeneous. *E. coli* strains isolated within the same indigenous population, and across different populations (from different villages), showed highly variable genotypes. Moreover, genetic diversity could be seen in *E. coli* strains isolated from the same individual less frequent than finding clonal strains. This observation is consistent with previous notion on the presence of resident and transient *E. coli *strains within the host’s gut in addition to the predominant clone (26). When compared across different locations, *E. coli *strains colonizing the indigenous population from Kampung Gurney at Hulu Yam showed a greater genetic homogeneity. The sharing of genetically similar *E. coli *clones between different individuals could be attributed to environmental factors and the diet of their hosts (26). Furthermore, the four VTEC strains identified in this study were found only in residents of Kampung Gurney. The prevalence of VTEC in one population over the other could be explained by the coincidental evolution hypothesis, which proposes that virulence factors might be a result of local adaptations to different ecological niches (27). Moreover, the carriage of two genetically distinct VTEC strains by a single individual residing in Kampung Gurney suggested that such adaptations were not exclusively found in a single *E. coli *lineage. 

Additionally, there was a report made by Liu* et al.* (2016) regarding the possibility that *mcr-1*-positive *E. coli* had spread to Malaysia (14). They made this assumption as they noted that five strains of *E. coli* containing *mcr-1*-like genes were submitted to the European Molecular Biology Laboratory (https://www.embl.de/). Prompted by that report, we have decided to investigate the prevalence of the *mcr-1* gene among *E. coli* isolates that were isolated from indigenous individuals in Malaysia. Liu and colleagues proposed that the colistin resistance gene, *mcr-1,* might have originated from animals and had subsequently spread to humans due to the lop-sided distribution of *mcr-1-*positive samples from animals and humans (14). In China, colistin had been widely used in the veterinary industry. However, the usage of colistin in the veterinary industry in Malaysia is relatively scarce. Nonetheless, Ong* et al.* have reported detection of three zoonotic *E. coli *strains that were resistant to colistin in the early 2010s (18).

Antibiotics play an essential role in curing illnesses associated with infectious diseases. However, selective pressure exerted by misuse and overuse of antibiotics has been the main reason that caused the emergence and spread of AMR traits among commensal and pathogenic bacteria. A study reported that based on surveillance data, *E. coli* resistance is consistently highest for antibiotics that have been used regularly in human and veterinary medicine (28). Resistance to sulfonamides has been observed in *E. coli* strains since 1950 and high prevalence of sulfonamides resistance was often associated with the presence and transmission of AMR genes *sul1* and *sul2* (28). The relatively high rate of resistance to AMP and SXT among the commensal *E. coli *strains in this study could be a result of the overuse of these antibiotics in primary care settings in Malaysia. Previous studies have shown that penicillin antibiotics including AMP, SXT, and CIP are among the most prescribed antibiotics for the empirical treatment of common bacterial infections in Malaysia, such as upper respiratory and urinary tract infections (29, 30).

## Conclusion

*E. coli *strains found in the intestines of indigenous populations in Malaysia were genetically diverse and remained largely susceptible to antibiotics. Asymptomatic carriage of VTEC has been detected among indigenous individuals. We believe that this study may provide foundational information for surveillance of the intestinal carriage of VTEC and AMR patterns of commensal *E. coli* among underprivileged indigenous individuals in Malaysia. 

## Authors’ Contributions

CSJT and KHC Conceived and designed the study protocol. YALL and RN Collected the study samples. VM and STN Performed the laboratory tests. CSJT, KHC, YALL, and RN Carried out the sample data acquisition. VM and STN Analyzed data and interpreted results. VM and STN did the literature search. CSJT Supervised the laboratory work and data analysis. All authors participated in drafting the article. VM and STN wrote the final form. All authors have approved the final manuscript for publication.

## Data Availability Statement

The original contributions generated for this study are included in the article/supplementary material, further inquiries can be directed to the corresponding author/s.

## Conflicts of Interest

The authors declare that they have no conflicts of interest.
